# Normothermic Machine Preservation of the Liver: State of the Art

**DOI:** 10.1007/s40472-018-0186-9

**Published:** 2018-02-27

**Authors:** Carlo D. L. Ceresa, David Nasralla, Wayel Jassem

**Affiliations:** 1 Nuffield Department of Surgical Sciences, University of Oxford, Oxford, UK; 2 Institute of Liver Studies, King’s College Hospital, London, UK

**Keywords:** Normothermic machine perfusion, Liver transplantation, Ischaemia reperfusion injury, Organ utilisation

## Abstract

**Purpose of Review:**

This review aims to introduce the concept of normothermic machine perfusion (NMP) and its role in liver transplantation. By discussing results from recent clinical studies and highlighting the potential opportunities provided by this technology, we aim to provide a greater insight into NMP and the role it can play to enhance liver transplantation.

**Recent Findings:**

NMP has recently been shown to be both safe and feasible in liver transplantation and has also demonstrated its superiority to traditional cold storage in terms of early biochemical liver function. Through the ability to perform a viability assessment during preservation and extend preservation times, it is likely that an increase in organ utilisation will follow. NMP may facilitate the enhanced preservation with improved outcomes from donors after cardiac death and steatotic livers. Furthermore, it provides the exciting potential for liver-directed therapeutic interventions.

**Summary:**

Evidence to date suggests that NMP facilitates the enhanced preservation of liver grafts with improved early post-transplant outcomes. The key role for this technology is to increase the number and quality of liver grafts available for transplantation and to reduce waiting list deaths.

## Introduction

The maintenance of solid organs ex vivo in a functioning state is a far from novel concept. In 1812, Le Gallois wrote that *“if one could substitute for the heart a kind of injection of arterial blood, either naturally or artificially, one would succeed easily in maintaining alive indefinitely any part of the body whatsoever”* [1]*.* However, it wasn’t until over 100 years later, in 1935 when Alexis Carell and Charles Lindbergh demonstrated the viability of abdominal organs perfused with oxygenated serum at normothermia for several days [2]. Thomas Starzl pursued this work further in the 1960s and indeed the first successful human liver transplants were performed following the pretreatment of livers with machine perfusion of diluted oxygenated blood [3]. In the subsequent years, interest in normothermic machine perfusion (NMP) reduced, largely due to the advent of cold preservation solutions which were less expensive and facilitated the transportation of organs from deceased donors [4]. However, in recent years, there has been a resurgence of interest in NMP. This is largely due to the need to expand the donor pool by successfully transplanting high risk or “marginal organs”. In this article, we will focus on NMP’s role in liver preservation and transplantation.

## The Dilemma Facing Liver Transplantation

The potential of liver transplantation to save the lives of patients with end-stage liver disease is limited by a shortage of donor organs. There are insufficient numbers of deceased donor livers available to meet waiting list demands and as a result, many patients die before they could benefit from a life-saving transplant. In the UK, the number of patients listed for a liver transplant has almost doubled in the last 10 years and as a result of the donor organ shortage, 15–20% of patients die whilst awaiting transplantation [5]. This concerning waiting list mortality rate is comparable to other regions [6, 7]. Although living donation can help to increase the donor pool, this is not available in every country and does not help the numerous patients without a suitable living donor. Furthermore, concerns are frequently raised about the risks of serious complications and donor mortality associated with the procedure. In many countries, therefore, greater focus has been directed to increasing utilisation of deceased donor livers. This involves pushing the boundaries of liver acceptance criteria, with a greater number of high risk or *marginal* livers being considered for transplantation. A marginal liver is defined as an organ with an increased risk of primary graft dysfunction or non-function, subjecting the recipient to increase morbidity and mortality risk. In recent years, the body of evidence demonstrating the successful transplantation of marginal livers has grown although there is still an accepted increased risk of complications such as primary non-function (PNF) and ischaemic cholangiopathy [8, 9].

## Features of a Marginal Liver

A liver is deemed to be marginal when there are certain donor and preservation characteristics which are known to increase the risk of post-transplant graft failure. All transplanted organs are subjected to ischaemia-reperfusion injury (IRI); a cascade of events initiated when an organ, previously deprived of oxygen, undergoes reperfusion. This occurs due to an efflux of toxic cellular products formed during ischaemia and triggers a profound inflammatory immune response, resulting in hepatocellular injury. Marginal livers have a lower tolerance of hypoxia, culminating in a more severe reperfusion injury. Furthermore, the resilience of a marginal organ to recover from the physiological and metabolic traumas of brain death, organ procurement, preservation and implantation is diminished. Livers considered marginal include those from older donors, donors after circulatory death (DCD) and livers which have a significant amount of intracellular fat deposition (hepatic steatosis). These will be discussed in more detail later in the text.

In recent years, there has been a shift in the type of donor and quality of liver procured and considered for implantation by transplant teams. Population dynamics has resulted in a change of donor characteristics to older donors with more comorbidities [10], and increasing levels of obesity will result in more steatotic livers [11]. An increase of marginal livers in the donor pool is therefore inevitable, and measures to salvage these organs for successful transplantation are of the utmost importance.

## Normothermic Machine Perfusion

**Fig. 1:**
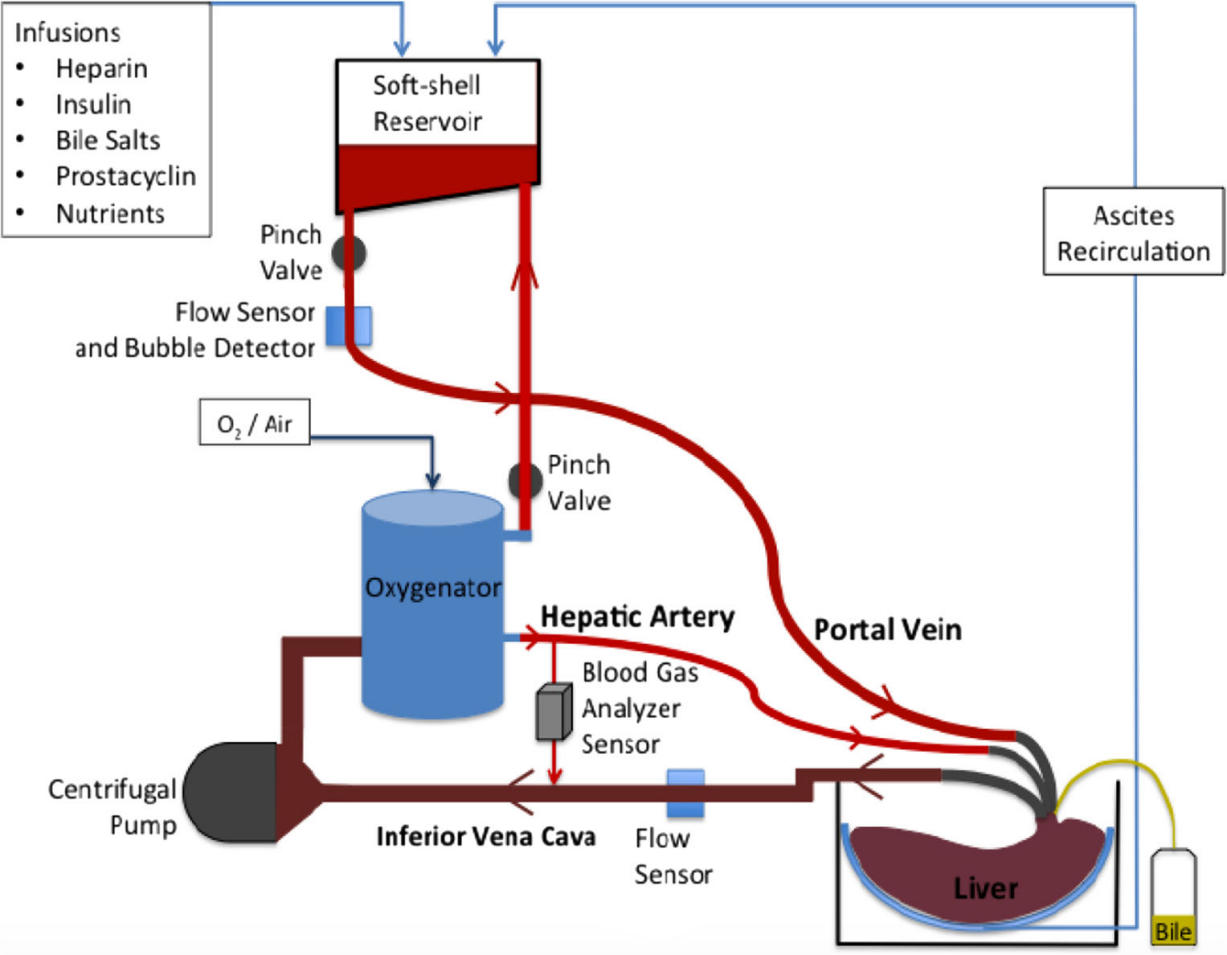
Schematic representation of OrganOx metra circuit

NMP aims to recapitulate an environment that mimics the human body, providing the liver with oxygen and nutrition at 37 °C. Several NMP circuits have been described [12–15] and have been constructed using components primarily developed for use in cardiopulmonary bypass. Principal constituents include a blood reservoir, pump(s) (some circuits comprise two pumps, representing portal venous and hepatic arterial flow), an oxygenator and heat exchanger. Currently, the devices which are either commercially available or being used in clinical trials include the following: the OrganOx *metra* (OrganOx Ltd., Oxford, UK), the Liver Assist (Organ Assist, Gronigen, the Netherlands), OCS™ Liver System (Transmedics, Andover, Massachusetts) and the Cleveland NMP circuit (Cleveland Clinic, Cleveland, Ohio). These devices mainly differ in the cannulation method (closed circuit vs open drainage), arterial flow (pulsatile vs continuous), portability and degrees of automation (pertaining to vascular pressures, flow rates and blood gas regulation). In terms of normothermic preservation, most published clinical outcome data and expertise lie with the OrganOx *metra* (OrganOx Ltd., Oxford, UK) device. This technology uses a normothermic suspension of red cells in a colloid to perfuse the liver in a fully cannulated system. Briefly, the perfusate is pumped out of the inferior vena cava using a centrifugal pump, heated and oxygenated. It is then diverted either to the hepatic artery through a high-pressure, low-flow system or to the soft-shell reservoir which feeds the portal vein via a high-flow, low-pressure system. Constant blood gas analysis enables monitoring and control of pO_2_ and pCO_2_ levels, facilitating maintenance of acid-base homeostasis. Continuous infusions ensure sufficient vasodilation, protection against coagulation and the provision of an environment that enables near-physiological metabolic and synthetic liver function [13]. A schematic representation of the OrganOx *metra* circuit is shown in Fig. 1.

NMP technology has been extensively tested in the non-clinical setting and was initially shown to facilitate the extended preservation of porcine livers which maintained synthetic and metabolic function for up to 72 h [16–18].

## Mechanism of Action

The main aim of NMP is to improve post-transplant outcomes through enhanced preservation which maintains functional integrity of the liver and reduces the deleterious impact of IRI. Static cold storage (SCS) is the current standard of care preservation method in solid organ transplantation, and it involves cooling the liver to 4 °C using a specialised preservation solution which aims to maintain structural and functional integrity of the liver so as to promote liver function at reperfusion. SCS contributes to IRI via a “two-hit” hypothesis. The first “hit” and ischaemic injury occur during in situ cold perfusion where organs are flushed at ice-cold temperature with preservation solution. Cessation of blood flow to the liver prevents the supply of oxygen, nutrients and co-factors but does not stop metabolic activity completely. It is merely slowed to 1.5- to 2-fold for every 10 °C drop in temperature. Anaerobic metabolism ensues, resulting in the accumulation of metabolic waste products, including breakdown products of adenosine triphosphate (ATP) [19]. Cell swelling and lysis occur due to ATP depletion which causes a loss of transcellular electrolyte gradients, influx of free calcium and subsequent activation of phospholipases [20]. The second “hit” or reperfusion injury results from the efflux of toxic molecules formed from metabolic products of ischaemia [21] and a profound inflammatory immune response that involves both direct and indirect cytotoxic mechanisms [22].

NMP’s superiority over SCS in terms of synthetic and metabolic liver function and post-transplant survival has been shown in large animal models [13, 14] through evidence of ex vivo bile production, reduction of markers of cell injury and post-transplant survival. However, the mechanism(s) supporting the beneficial effects of NMP have not yet been fully explained. It is hypothesised that perfusion helps maintain a healthy endothelium and replenish adenosine triphosphate (ATP). This was confirmed in a porcine model where tissue ATP content was restored to 80% of the original level after 4 h of NMP preceded by sequential warm and cold ischaemia [23]. More recently, human studies have demonstrated histological evidence of glycogen repletion during NMP which is important in supplying glucose for ATP generation [24••]. In order to further exploit and maximise NMP’s potential, a sound understanding of the injury and repair pathways affected during preservation will be important. A difference in gene expression has also been shown between transplanted human NMP and SCS livers. Comparing gene expression between pre- and post-reperfusion biopsies, genes upregulated after NMP were mainly those involved in tissue regeneration, tissue growth/repair and those involved in control of inflammation. In contrast, upregulated genes in SCS were mainly those implicated in inflammation, apoptosis and activation of coagulation [25]. Further studies need to be carried out to further explore NMP’s mechanism of action which will undoubtedly allow clinicians and scientist to further exploit the technology to improve outcomes.

## Clinical Experience of NMP

In recent years, clinical outcomes from livers preserved via NMP have been reported. The first was a phase I study by Ravikumar et al. which demonstrated the safety and feasibility of NMP [26••] using the OrganOx *metra* (OrganOx Ltd., Oxford, UK) device. NMP livers were preserved for a median of 9.3 h. There was evidence of stable hemodynamics, synthetic and metabolic function throughout all perfusions with maintenance of pH between 7.2 and 7.4. Bile production commenced after the first hour and was maintained, with an upward trend, throughout NMP. Outcomes were compared with cold-stored matched controls, and 30-day graft survival was similar in the two groups (100% NMP vs. 97.5% control, *p* = 1.00). However, livers preserved via NMP had a significantly lower peak serum aspartate aminotransferase (AST) in the first 7 days post-transplant (417 IU [84–4681]) versus (902 IU [218–8786], *p* = 0.03). A number of studies have demonstrated a relationship between peak serum AST in the early post-transplant period and patient survival, graft survival, early graft dysfunction and primary non-function following liver transplantation [27, 28]. This improvement in early biochemical liver function may represent a reduced preservation injury. Following on from this study, the Toronto and Edmonton groups have also published results from phase I studies using the same NMP device, transplanting 10 and 9 NMP livers, respectively [29, 30]. Both groups showed the technology to be safe and feasible for the entire preservation period, from retrieval to transplantation, including transportation. Selzner and colleagues demonstrated a non-significant reduction in peak serum AST in the NMP-preserved livers compared to retrospective SCS controls, but this finding was not achieved by Bral and colleagues, in whose study there was a non-significant increase in the median peak serum AST in the NMP cohort. None of the studies was either randomised or powered to demonstrate a difference in outcomes. Across these three studies, only one graft was lost during preservation. This was due to an occult twist in the portal vein which was obscured within the hepatic hilum, compromising perfusion of the liver [30].

The first randomised controlled trial comparing the efficacy of NMP compared to SCS in adult liver transplantation has recently been completed by the Consortium for Organ Preservation in Europe (COPE; www.cope-eu.org). Two hundred and twenty-two livers were transplanted as part of the study (121 NMP, 101 SCS), and data from a published abstract [31] demonstrated a significant reduction in peak serum AST in the NMP group (974 IU/L SCS vs 485 IU/L NMP; *p* < 0.001). There was also a significant reduction in early allograft dysfunction (EAD), another surrogate marker of graft outcome, in livers preserved via NMP (29.9% SCS vs 12.6% NMP; *p* = 0.002). Another important finding was that of a significantly reduced rate of liver discard in organs randomised to NMP (32 SCS vs 16 NMP; *p* = 0.01). Although, to date, no trial has shown an improvement in patient or graft survival or reduction in biliary complications, it is notable that trials with much larger numbers and longer term follow-up would be required to test these outcomes.

## Specific Benefits of NMP

### Donors after Circulatory Death

DCD organs constitute a high and increasing proportion of marginal grafts. In the UK, 42% of the donor pool and 21% of transplants are made up of DCD livers [32]. Although in other regions such as the US and Euro-transplant zones, the number of DCD liver transplants is considerably less; NMP may provide the ideal platform to increase liver utilisation from this type of donor. Despite an increased effort to transplant DCD livers, unfortunately, graft loss and recipient mortality have been shown to be almost twice as high as with DBD livers [33]. During a DBD retrieval, although there are physiological changes as a result of brain death, there is minimal functional warm ischaemia time (FWIT) and oxygenation is maintained until the organ is perfused with cold preservation solution. During retrieval of a DCD organ, the FWIT is deemed to have started when the systolic blood pressure has a sustained fall below 50 mmHg and extends up to the onset of cold in situ perfusion [34]. The duration of the FWIT is an important determinant of outcome and a recent large single centre study reported an increasing FWIT’s association with recipient complications, specifically ischaemic cholangiopathy and graft failure [35]. To this end, a FWIT of less than 20 min is advised [36–38]. These criteria are arbitrary and the decision as to whether to proceed is multifactorial; as a result, a number of livers are retrieved from DCDs that are, on consideration of many factors, then declined.

NMP’s benefits have been demonstrated in porcine DCD transplantation models [13, 14]. Schon et al. [14] and Brockmann et al. [13] both demonstrated enhanced survival and synthetic liver function in a porcine model of DCD transplantation. Brockmann et al. investigated extended preservation times and demonstrated superior outcomes in livers preserved for 20 h compared to 4 h or cold storage. This highlights the potential of NMP in eliciting organ repair. It is important to mention, however, that both these groups did not demonstrate a functional or survival benefit of NMP in the DBD cohort when livers were not subjected to warm ischaemia prior to preservation. An exciting finding from the recent COPE RCT was the greater improvement in early biochemical function observed with NMP livers in the DCD cohort (*p* = 0.02) [31]. These data suggest that NMP has a role in reversing energy depletion and the immediate effects of warm ischaemia; this may be a particularly important finding when DCD liver utilisation remains low.

As previously mentioned, a major cause of morbidity and graft loss after DCD liver transplantation is ischaemic cholangiopathy which affects around 16% of transplanted livers [39]. In a porcine DCD model, Liu et al. demonstrated biliary epithelial regeneration in NMP livers and a significant improvement in histological biliary scoring when compared to cold-stored grafts [40]. If translated into clinical transplantation, this finding would have an enormous beneficial impact.

### Steatotic Livers

The utilisation of steatotic livers is limited by a greatly increased susceptibility to IRI [41]; ~ 1000 steatotic livers are discarded each year in the USA [42]. One large-scale study demonstrated macrovesicular steatosis involving more than 30% of hepatocytes decreased graft survival post-transplant by 71% [43]. With increasing levels of obesity and non-alcoholic fatty liver disease (NAFLD) worldwide, an increase of steatotic livers in the donor pool is inevitable. Animal models have suggested that NMP has the potential to facilitate the safe and reliable transplantation of steatotic livers through enhanced preservation and de-fatting. Jamieson et al. explored the effects of NMP using a porcine model of hepatic steatosis, and NMP maintained physiological function comparable to normal, non-steatotic livers. It was found that the degree of steatosis was reduced with a decrease in the total amount of lipid droplets and average lipid deposit size [44]. Nagrath et al. perfused steatotic rat livers for 3 h with a “defatting cocktail” in an oxygenated, normothermic system and observed a 65% reduction in hepatic triglyceride content [45]. This could be explained through the potentiation of fatty acid esterification and oxidation during perfusion. To date, there is no published data on the effect of NMP on human steatotic livers; an exciting area for future research.

### Extended Preservation Times

Current cold storage with a preservation solution facilitates the storage and transportation of a liver for up to 16 h before transplantation [46]. However, over the last 10 years in the UK, preservation times have been considerably lower with a median of 8 h for DBD donors and 7 h for DCD donors [32]. This situation in the USA is similar, with median preservation time in the region of 8 h [43]. NMP devices have the benefit of extending the preservation period for up to 24 h. NMP has also been used in combination with cold storage in the context of organ reconditioning. A recent report by Watson et al. described the successful transplantation of a liver which had been preserved for a total of 26 h, of which 8.5 h were normothermic, using the Liver Assist device (Organ Assist, Gronigen, the Netherlands) [47]. Extended preservation times allow a more organised and structured approach to transplantation by improving utilisation of the operating room and elective lists, arranging appropriate staffing levels and can also facilitate the medical optimization of the recipient. It could also provide a critical time period which may be required for organ enhancement through liver-directed therapeutic interventions [48].

### Viability Assessment

The ability to predict post-transplant outcome is an attractive prospect and could improve organ utilisation. Over the course of the last 10 years in the UK, the number of livers retrieved but not transplanted has doubled from 8.2 to 16.6% [32]. The situation is slightly worse in the USA, with the latest report of the Organ Procurement and Transplant Network revealing that only 78% of potential donor livers were transplanted [6]. Livers tend to be discarded following an assessment of donor characteristics and risk as well as the gross appearance of the organ [49, 50]. However, the lack of objective predictors of function will invariably result in viable organs being turned down. NMP provides an opportunity to make an objective assessment on the liver’s function during preservation and could potentially increase our ability to reliably predict post-transplant outcomes. In a recent series reported by Mergental et al., six livers which had been rejected by all UK transplant centres were perfused with the Liver Assist device (Organ Assist, Gronigen, the Netherlands) [24••]. They developed viability criteria (to be fulfilled within 3 h of perfusion) on which to base the liver’s suitability for transplantation. Their criteria include the liver’s ability to clear lactate, produce bile, maintain acid/base homeostasis and stable pressure and flow dynamics as well as a healthy physical appearance of the graft. Five out of the six livers fulfilled the criteria and were subsequently transplanted. At a median 7 (range 6–19) months follow-up, all recipients remained well with functioning grafts. Other groups have suggested the importance of perfusate lactate and transaminases as well as bile production in the functional assessment of liver viability [12]. Watson et al. also published their experience of transplanting livers which had been declined by other UK liver transplant centres, following NMP and assessing organ quality and function [51•]. They observed that preserving livers at physiological oxygen tensions resulted in a reduced incidence of post-reperfusion syndrome and suggested the importance of biliary pH in predicting post-transplant cholangiopathy. It is important to mention that none of these viability markers are validated, and the small numbers make robust conclusions difficult. Larger clinical trials need to be conducted before validated markers can be identified. Even if the sole benefit of NMP was shown to be improved organ utilisation with superior early biochemical function when compared to SCS, this would have a great impact on reducing the number of deaths on the waiting list.

### Drug Delivery

NMP provides a potential platform to treat the liver ex vivo during the preservation period. This is unique to NMP, as opposed to other machine perfusion techniques, as active metabolism permits graft intervention and modification during preservation. This could be applied in the context of de-fatting steatotic livers, as previously mentioned [44, 45]. Another exciting potential is that of immunomodulation to induce tolerance of the liver. This has already been demonstrated by infusing adenoviruses expressing CTLA4Ig directly into the portal circulation of a rat [52] and could be applied to the perfused liver. It has also been suggested that NMP could be used to deliver gene therapies such as myr-Akt in an attempt to provide cytoprotection against ischaemia reperfusion injury [53].

## Conclusion

Normothermic machine perfusion has heralded a new era in liver transplantation and organ preservation. It is hoped that with this new technology, more livers can be transplanted with improved outcomes and that fewer lives will be lost on the waiting list. More clinical studies are required to assess its true clinical impact as well as exploit its true potential in organ regeneration and enhancement.
